# Anti-Epidermal Growth Factor Receptor Gene Therapy for Glioblastoma

**DOI:** 10.1371/journal.pone.0162978

**Published:** 2016-10-06

**Authors:** Martin J. Hicks, Maria J. Chiuchiolo, Douglas Ballon, Jonathan P. Dyke, Eric Aronowitz, Kosuke Funato, Viviane Tabar, David Havlicek, Fan Fan, Dolan Sondhi, Stephen M. Kaminsky, Ronald G. Crystal

**Affiliations:** 1 Department of Genetic Medicine, Weill Cornell Medical College, New York, New York, United States of America; 2 Department of Radiology, Weill Cornell Medical College, New York, New York, United States of America; 3 Department of Neurosurgery, Memorial Sloan-Kettering Cancer Institute, New York, NY, United States of America; Swedish Neuroscience Institute, UNITED STATES

## Abstract

Glioblastoma multiforme (GBM) is the most common and aggressive primary intracranial brain tumor in adults with a mean survival of 14 to 15 months. Aberrant activation of the epidermal growth factor receptor (EGFR) plays a significant role in GBM progression, with amplification or overexpression of EGFR in 60% of GBM tumors. To target EGFR expressed by GBM, we have developed a strategy to deliver the coding sequence for cetuximab, an anti-EGFR antibody, directly to the CNS using an adeno-associated virus serotype rh.10 gene transfer vector. The data demonstrates that single, local delivery of an anti-EGFR antibody by an AAVrh.10 vector coding for cetuximab (AAVrh.10Cetmab) reduces GBM tumor growth and increases survival in xenograft mouse models of a human GBM EGFR-expressing cell line and patient-derived GBM. AAVrh10.CetMab-treated mice displayed a reduction in cachexia, a significant decrease in tumor volume and a prolonged survival following therapy. Adeno-associated-directed delivery of a gene encoding a therapeutic anti-EGFR monoclonal antibody may be an effective strategy to treat GBM.

## Introduction

Glioblastoma multiforme (GBM), the most common and aggressive primary intracranial brain tumor in adults, has a mean survival of 14 to 15 months following diagnosis [1–5]. The current standard intervention involves surgery followed by radiation and chemotherapy [3, 6]. Due to the diffuse, invasive nature of the disease, complete resection of the tumor is difficult to achieve, and recurrences at the surgical margins and other areas of the brain are common, even after repeated surgery, radiation and chemotherapy [7, 8].

Aberrant activation of the epidermal growth factor receptor (EGFR), a tyrosine kinase receptor that binds ligands of the epidermal growth factor family, plays an important role in GBM, where dysregulated expression or activity is associated with tumor development, progression, metastatic spread, and decreased survival [9–11]. Amplification or overexpression of EGFR is present in 60% of GBM tumors, with more than 40% of those tumors carrying the EGFR variant III mutant (EGFRvIII), a mutation that results in a truncated receptor that causes constitutive signaling pathway activation [9, 10, 12, 13]. One anti-EGFR targeting approach involves the systemic delivery of an anti-EGFR monoclonal antibody. For example, cetuximab (Erbitux; ImClone Systems), a recombinant human/mouse chimeric monoclonal antibody which can inhibit EGFR and EGFRvIII by interfering with ligand binding, targets the receptor for internalization and degradation [14–17]. Systemic administration of cetuximab reduces cellular proliferation in a variety of cancer models, and is well tolerated when given intravenously to patients with recurrent GBM [15, 18]. However, anti-EGFR efficacy with an anti-EGFR monoclonal administered systemically is limited by the blood-brain barrier, where <0.5% of circulating antibodies reach the brain, and by the need for repeated administration of the therapeutic due to its short half-life in serum [16, 19–23].

To circumvent these limitations on efficacy, we have developed a strategy to deliver the coding sequence for an anti-EGFR antibody directly to the CNS via an adeno-associated virus serotype rh.10 (AAVrh.10) gene transfer vector. The objective is to bypass the blood-brain barrier by producing local, sustained therapeutic antibody levels in the CNS. When administered directly to the CNS, the AAVrh.10 vector mediates high levels of protein expression, particularly in neurons, and has been shown to be safe in clinical studies of CNS gene transfer [24–26] (clinicaltrials.gov, NCT01161576). In the present study we demonstrate that single, local delivery of an anti-EGFR antibody by an AAVrh.10-derived vector (AAVrh.10CetMab) reduces GBM tumor growth and increases survival in xenograft mouse models of an EGFR-expressing GBM cell line and patient-derived GBM cells.

## Methods

### AAVrh.10CetMab Vector

The AAVrh.10CetMab vector is derived from the rhesus macaque AAV rh.10 capsid pseudotyped with AAV2 inverted terminal repeats on both the 5’ and 3’ ends of the anti-EGFR antibody expression cassette. The expression cassette was designed (5’ to 3’) with the cytomegalovirus promoter containing the chicken–β-actin enhancer (CAG), the sequence coding for the cetuximab monoclonal heavy chain (IgG1), the 4-amino-acid furin cleavage site, the 24-amino-acid self-cleaving 2A peptide from *Thosea asigna* virus (*Ta*v), the cetuximab light chain (κ chain), and the rabbit β-globin polyadenylation signal [24, 27, 28].

The AAVrh.10CetMab vector was generated by transfection using Polyfect (Qiagen) or PEI MAX (Polysciences, Inc.) and human embryonic kidney 293T cells using the two plasmids, pAAVαEGFR, and pPAKMArh.10.) pAAVαEGFR is an expression plasmid containing (5′ to 3′) the AAV2 5′-inverted terminal repeat including packaging signal (ψ), the expression cassette of the chimeric (mouse and human) anti-EGFR monoclonal antibody cetuximab and the AAV2 3′-inverted terminal repeat. pPAKMArh.10 is a helper and packaging plasmid that expresses the AAV Rep proteins derived from AAV2 required for vector replication, the AAVrh.10 viral structural (Cap) proteins VP1, 2 and 3, and provides Ad helper functions of E2, E4 and V_A_ RNA [24, 29]. At 72 hr after transfection, the cells were harvested and lysed by four repeated cycles of freeze and thaw, and the homogenate was clarified by centrifugation to remove cell debris and collect the crude viral lysate. AAVrh.10CetMab was purified by iodixanol gradient and QHP anion exchange chromatography, concentrated with an Amicon Ultra-15 100K centrifugal filter device (Millipore) and stored in PBS, pH 7.4, -80°C. Vector genome titers were determined by TaqMan quantitative PCR using a cytomegalovirus promoter-specific primer–probe set (Applied Biosystems, Grand Island, NJ). To verify AAVrh.10CetMab-directed expression of cetuximab, HEK293Torf6 cells were infected with AAVrh.10CetMab at 2 x 10^5^ genome copies per cell (or mock infected), supernatant was harvested 72 hr later and immunoglobulin was purified with protein G sepharose. Cetuximab expression was evaluated by SDS-PAGE and Western analysis with a sheep anti-human IgG heavy chain and light chain secondary antibody (Sigma) and an enhanced chemiluminescence reagent (Amersham).

### Mouse GBM Models

U87MG (American Type Culture Collection, Manassas, VA) glioblastoma cells were cultured in Eagle's Minimum Essential Mediumwith fetal bovine serum (10%). To generate a GBM cell line expressing high levels of EGFR, the retroviral expression vector pMFG vector was used to generate replication-defective viruses with sequence coding for the EGFR along with a GFP reporter [30, 31]. The viral supernatants were used to infect the U87MG cells, and U87MG cells expressing wt-EGFR-GFP protein were passaged to create U87MG:EGFR cells.

For the model with primary human glioblastoma cells, low passage primary human glioblastoma cells (MSK0709) were cultured in DMEM/F12 with epidermal growth factor (EGF, 20 ng/ml),basic fibroblast growth factor (bFGF, 20 ng/ml) and N2 supplement (Invitrogen) on 10 cm plates (BD Biosciences, San Jose, CA) coated with poly-L-ornithine (1:1000 of 15 mg/ml), laminin (1:250 of 1 mg/ml, R&D Systems, Minneapolis, MN) and fibronectin (1:500 of 1 mg/ml, BD Biosciences). The derivation of the line was carried out under an IRB-approved protocol at Memorial Sloan Kettering Cancer Center.

Based on the knowledge that administration of AAVrh.10 vectors express the gene coded by the expression cassette within 1 wk *in vivo* [32], and based on the hypothesis that the local expression of cetuximab would contribute to inhibition of growth of residual tumor cells that would persist after surgery, AAVrh.10CetMab or PBS (control) were administered to the CNS simultaneously with the tumor cells or 8 days or 3 wk after implantation of the tumor cells. All animal studies were conducted under protocols reviewed and approved by the Weill Cornell Institutional Animal Care and Use Committee. Female NOD/SCID immunodeficient mice, 6 to 8 wk old (Jackson, Bar Harbor, ME) were housed under pathogen-free conditions. At 7 to 10 wk of age the mice received 10^5^ U87MG:wtEGFR tumor cells (2 μl volume) plus 1011 genome copies (gc) AAVrh.10CetMab or PBS by direct CNS administration (2 μl volume). CNS administration of the tumor cells and the vector were administered stereotaxically to the lower striatum of the right hemisphere (A/P +1.0 mm, M/L ±1.0 mm, D/V—3.0 mm). The 10 μl syringe (Hamilton, Reno, NV) with a 26 g needle was lowered into position, and after 2 min the vector or PBS was delivered at a rate of 0.5 μl/min. The needle remained in place for 2 min after administration, and was then withdrawn (1 mm), remained for 1 min, and then gradually withdrawn over the course of 1 min.

For the U87MG:wtEGFR xenograft model treated 8 days after tumor administration, 10^5^ U87MG:wtEGFR cells were administered in a volume of 2 μl, followed 8 days later by 10^11^ gc AAVrh.10CetMab or PBS in a volume of 2 μl. For the 0709 low passage primary cells, 10^5^ cells were administered in a total volume of 3 μl. After 3 wk, mice were administered 10^11^ gc AAVrh.10CetMab or PBS control in a volume of 2 μl.

All mice were monitored for signs of neurological impairment, cachexia, or significant loss of weight (decrease in 1/3 adult body weight) and sacrificed at the time point indicated. The weight of mice was measured at day 0, and upon signs of cachexia, on a daily basis between days 21 through 28.

### Regional Expression of Cetuximab

Samples of brain tissue were collected after perfusion with cold phosphate buffered saline (PBS, pH 7.4). Three coronal sections (equidistance anterior to posterior) of mouse brain were divided into 2 hemispheres. The localized expression of cetuximab antibody was determined by ELISA of the brain homogenate from each section. Wells of flat bottomed 96-well EIA/RIA plates (Corning, Corning, NY) were coated with 100 μl of 0.2 μg/ml EGFR (R&D Systems), in carbonate-buffer at pH 9.0 overnight at 4°C and then washed with 0.05% Tween 20 in PBS (PBS-Tween) and blocked with 5% dry milk in PBS for 30 min, 23°C. Serial dilutions of clarified homogenate were added to the 96-wells and incubated for 90 min, 23°C. The plates were washed 4 times with PBS-Tween and 100 μl of 1:2000 diluted goat anti-human IgG conjugated to horseradish peroxidase (Sigma-Aldrich, St. Louis, MO) in 1% dry milk in PBS, incubated for 90 min, 23°C. After 4 wash steps, peroxidase substrate (100 μl/well; Bio-Rad, Hercules, CA) was added to each well, incubated for 15 min, 23°C and the reaction was stopped with addition of 2% oxalic acid (100 μl/well). Absorbance was measured at 415 nm. Cetuximab antibody titers were calculated by interpolation of the log(OD)-log(dilution) with a cutoff value equal to twice the absorbance of background and converted to μg/ml based on standard curve with the cetuximab protein. Total protein levels were quantified by the bicinchoninic acid assay (Pierce Biotechnology, Rockford, IL).

### Magnetic Resonance Imaging

Magnetic Resonance Imaging was performed on a 7.0 Tesla 70/30 Bruker small animal MRI scanner (Bruker Biospin, Bilerica, MA) equipped with an additional small animal imaging gradient set (45 G/cm). Animals were imaged under isoflurane anesthesia (2% to initiate anesthesia, 1% for maintenance). A warming bed was used to maintain a constant body temperature and respiration was also monitored throughout the imaging procedure.

High-resolution imaging sequences were acquired following tail vein administration of the MRI contrast agent gadopentetate dimeglumine (Gd-DTPA; Berlex Laboratories; Wayne, NJ; 8 nmol/mouse in 40 μl volume). A T1-Weighted 2D FLASH sequence was used to visualize contrast enhancement with a repetition time of 357 ms and an echo time of 3.8 ms. T2-Weighted Turbo RARE sequences were also acquired with repetition and echo times of 2300 ms/48 ms, respectively, to detect edema. A 20 mm field of view and 256 x 256 matrix produced an image resolution of 78 μm x 78 μm x 500 μm with 20 matching axial slices. Tumor burden, tumor volume (ml) was assessed using T1 (Gd-DTPA enhanced) and T2 weighted sequences using IDL 8.1 custom coded algorithms (Exelis Visual; Boulder, CO) of the demarcated tumors.

### Statistics

Data are expressed as means ± standard error, and comparisons between groups were conducted by a one-tailed unpaired t-test. Comparisons between treatment groups at multiple time points were conducted by two-way analysis of variance (MedCalc Software, Ostend, Belgium). The survival data was generated using Kaplan-Meier survival plot and groups were compared using the Mantel-Cox test (GraphPad Software, Inc., La Jolla, CA).

## Results

**Fig 1:**
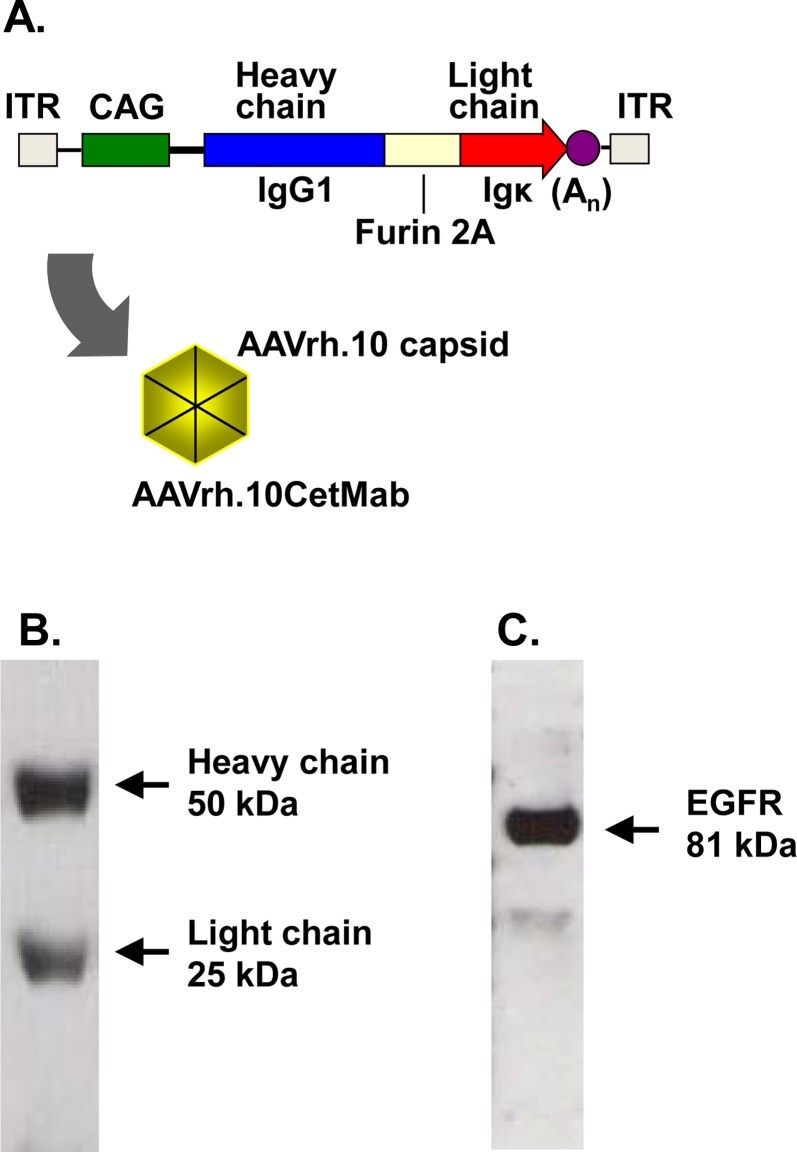
AAVrh.10- mediated expression of cetuximab. **A**. Design of the AAVrh.10CetMap vector expressing anti-EGFR (cetuximab). The antibody cDNA construct includes the heavy chain, a furin recognition cleavage site upstream of a 2A sequence (Furin 2A) and the light chain. The expression cassette includes the CAG promoter and the rabbit β-globin polyadenylation signal. **B**. Expression of cetuximab in supernatant of HEK293orf6 cells 72 hr after transfection with a plasmid expressing the cetuximab cDNA. Arrows indicate the expected size for heavy and light chains. **C.** Recognition of purified EGFR protein (1 μg) by cetuximab expressed in the supernatant of the HEK293orf6 transfected cells. The arrow shows the expected size for EGFR (81 kDa).

**Fig 2:**
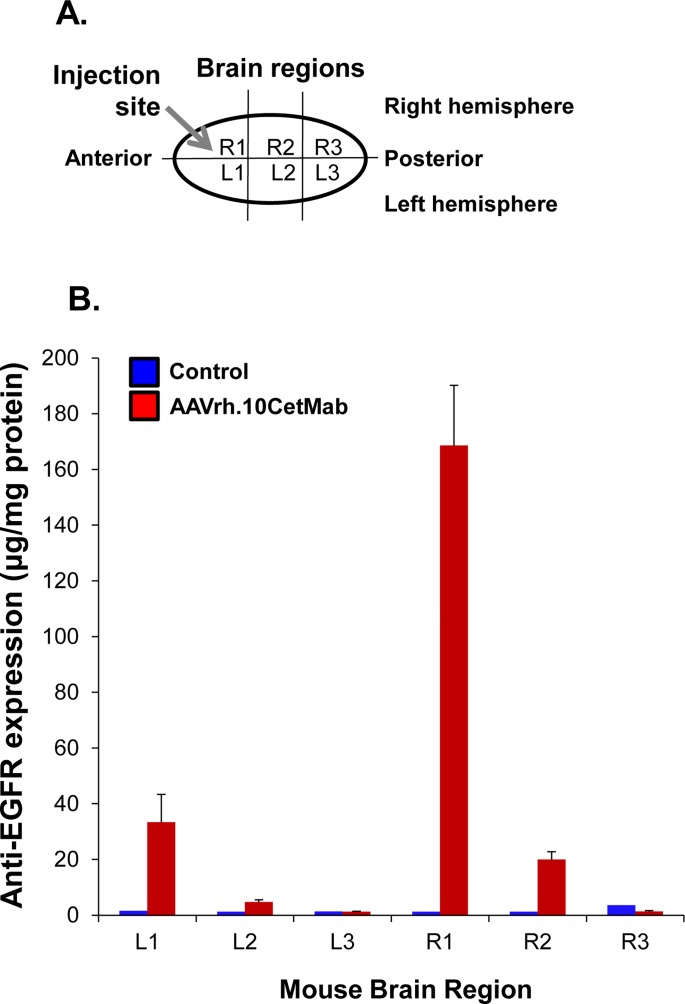
Cetuximab expression in the mouse brain after single administration of AAVrh.10CetMab. **A.** diagram of the mouse brain illustrating the site of vector administration and expression analysis. R = right; L = left. The vector was administered in region R1. **B.** Cetuximab expression in the mouse CNS. NOD/SCID mice (n = 4, male) received a single administration of 10^11^ genome copies (gc) of AAVrh.10CetMab. Cetuximab expression was quantified by ELISA in brain homogenates 3 wk after administration. Cetuximab levels were normalized per mg of tissue (mean ± standard error).

The AAVrh.10CetMab vector was designed using rhesus macaque serotype AAVrh.10 capsid proteins combined with a genome comprised of the inverted terminal repeats of AAV2 surrounding the anti-EGFR antibody expression cassette (Fig 1A). The AAVrh.10CetMab vector effectively generated the functional heavy and light chain of the chimeric anti-EGFR monoclonal antibody, cetuximab (Fig 1B) that binds to EGFR (Fig 1C). To assess the expression of cetuximab delivered by AAVrh.10CetMab *in vivo* in the CNS, the AAVrh.10CetMab vector was administered to the striatum of the right hemisphere (region R1) of the CNS (Fig 2A). Three wk after administration, the mouse brain was collected, segmented and the identified regions (R1, R2, R3, L1, L2 and L3) quantified for expression of cetuximab by ELISA standardized to total protein levels (Fig 2B). The targeted area of the caudate putamen, R1, showed the highest level of cetuximab expression (168±22 μg/ml), with about 4-fold less expression in the left anterior L1 region (34±10 μg/ml), and approximately 8-fold less in the mid-region of the right hemisphere, R2 (20±3 μg/ml; Fig 2B). Overall, the anti-tumor antibody showed high expression in the targeted region and negligible expression in the non-targeted left hemisphere and posterior region of the CNS.

**Fig 3:**
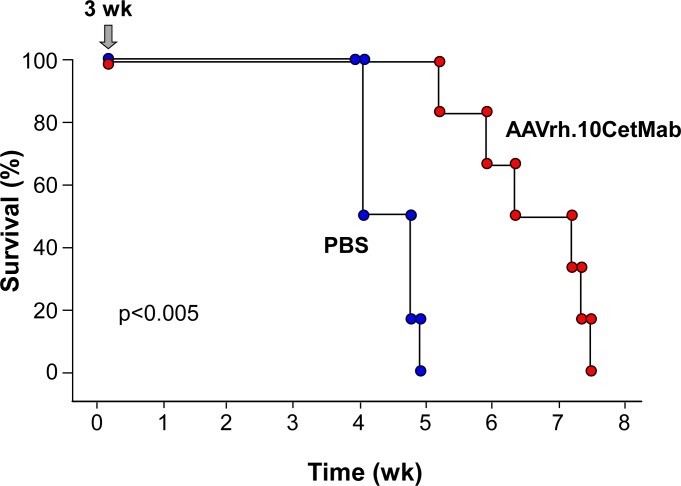
Survival of mice with U87MG:wtEGFR human glioblastoma xenografts treated with AAVrh.10Cetmab at the same time as tumor implantation. NOD/SCID mice (n = 6, male) received a single CNS administration of 10^11^ genome copies (gc) of AAVrh.10CetMab or PBS simultaneously with 10^5^ U87MG:wtEGFR glioblastoma cells. Arrow indicates the time of tumor implantation and treatment.

To mimic the characteristic overexpression of EGFR common in GBM, NOD/SCID mice were implanted with U87MG tumor cells engineered to overexpress the wild-type epidermal growth factor receptor (U87MG:wtEGFR). The AAVrh.10.CetMab vector was delivered simultaneously along with the U87MG:wtEGFR xenograft. The control group received U87MG:wtEGFR implant and PBS in place of the viral vector. During the course of the experiments, signs of cachexia were monitored and body weight assessed. On day 20, AAVrh.10CetMab-treated mice maintained weight (20.7±0.3 gm), while PBS treated mice exhibited a decrease in weight (18.9±0.5 gm, AAVrh.10CetMab-treated *vs* control, p<0.006). On day 28, a significant difference in weight loss was observed between the two groups. AAVrh.10CetMab-treated mice maintained the same weight (20.2±0.3 gm), while the non-treated control mice showed a further decrease in weight (17.3±1.2 gm, AAVrh.10CetMab treated *vs* control, p<0.008 at day 27; S1 Fig). Treatment with AAVrh.10CetMab increased the median survival time of mice with U87MG:wtEGFR GBM xenografts by 32% (n = 6, AAVrh.10.CetMab treated *vs* control, p<0.002; Fig 3).

**Fig 4:**
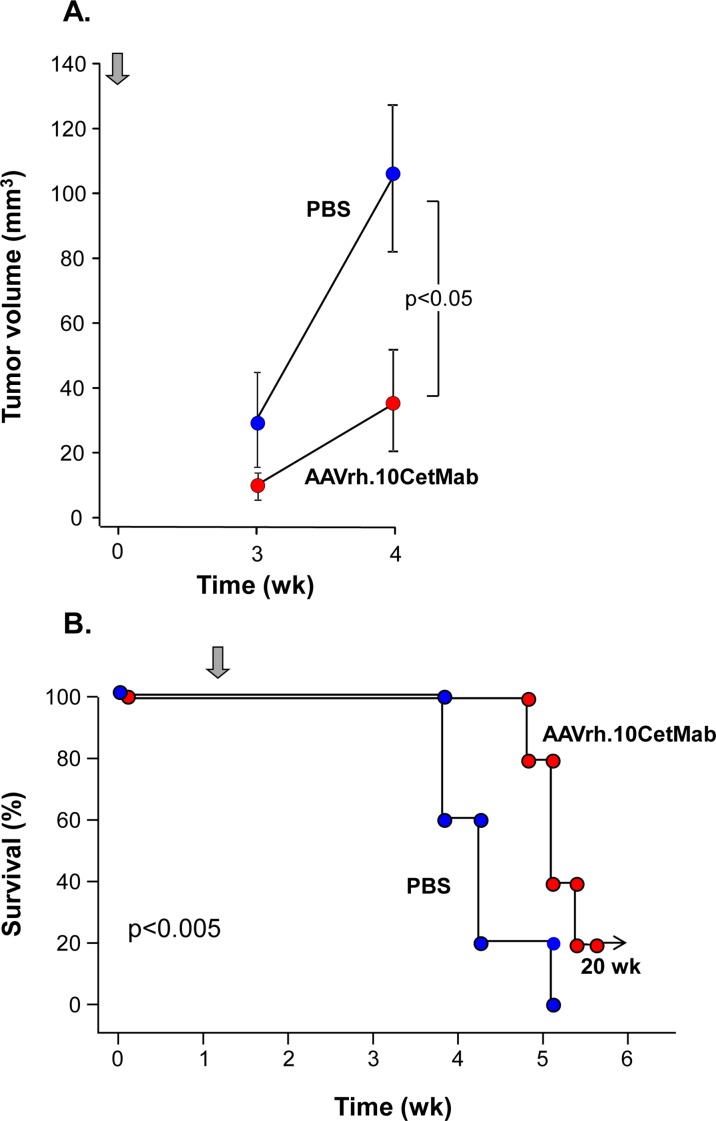
Treatment of mice with U87MG:wtEGFR human glioblastoma xenografts treated 8 days after xenograft implementation. **A.** Quantitative CNS MRI assessment of tumor volume. Male NOD/SCID mice received a CNS administration of 10^5^ U87MG:wtEGFR glioblastoma cells. Eight days after xenograft implant, mice received 10^11^ gc AAVrh.10Cetmab or PBS (n = 3 each group). Arrow indicates time of vector administration. MRI imaging of the tumors was carried out at 3 and 4 wk after treatment administration. Shown is quantitative assessment of tumor volume of PBS-treated control (n = 3) *vs* AAVrh.10CetMab-treated mice (n = 3). **B.** Survival. NOD/SCID mice (n = 10, male) received CNS administration of 10^5^ U87MG:wtEGFR glioblastoma cells. Eight days after xenograft implant, mice received 10^11^ gc AAVrh.10CetMab or PBS (n = 5 each group). Arrow indicates the time of treatment.

In the 8 day post-xenograft model of treatment of U87MG:wtEGFR GBM, at 3 wk no significant difference was observed in the AAVrh.10CetMab treated mice as compared to the PBS control. However, by wk 4 the AAVrh.10CetMab treated mice showed a significant 3-fold reduction in tumor volume as compared to PBS treated control mice (n = 3, AAVrh.10.CetMab post-xenograft treated *vs* control, p<0.05; Fig 4A; S2 Fig). All AAVrh.10.CetMab-treated mice that received the vector 8 days after the xenograft survived through wk 5 compared to only 20% survival in the PBS treated group (n = 5, AAVrh.10CetMab treated *vs* control, p<0.03; Fig 4B).

**Fig 5:**
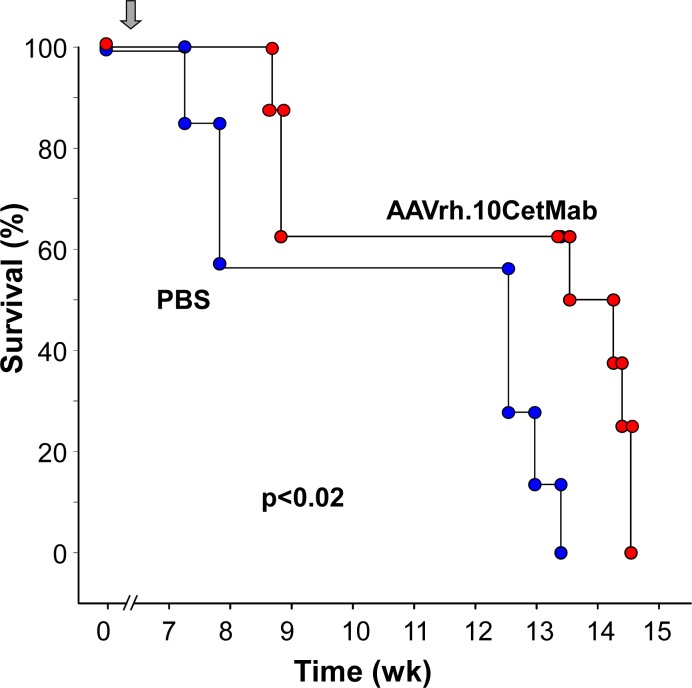
Survival of mice with patient-derived human glioblastoma xenograft treated with AAVrh.10CetMab. Male NOD/SCID mice received a CNS administration of 10^5^ primary glioblastoma cells isolated from a patient (isolate #0709). Three weeks after xenograft implant, mice received 10^11^ gc AAVrh.10CetMab or PBS (n = 7 each group) and survival was assessed as a function of time. Arrow indicates the time of treatment.

In the studies with primary GBM, NOD/SCID mice were implanted with GBM cells obtained from a recently derived patient tumor (and passaged briefly in serum-free media). Three wk post-xenograft, mice were administered the AAVrh.10.CetMab vector. In this model, survival was extended in the AAVrh.10.CetMab treated group, with a 60% increase survival through wk 13 compared to the PBS treated group (n = 7, AAVrh.10CetMab treated *vs* control, p<0.02; Fig 5).

## Discussion

Growth advantage of glioblastoma through EGFR overexpression and/or activation is a common characteristic of GBM, representing 60% of cases [4, 5, 7, 10, 13]. Current strategies to deliver an effective and persistent dose of anti-EGFR monoclonals to the GBM tumor microenvironment have not been successful, in part because the blood brain barrier severely limits the systemically administered anti-EGFR therapy from accessing the tumor microenvironment [4, 5, 16, 19, 20, 33, 34]. In the present study, we present a novel strategy to deliver to the CNS a gene that encodes a persistent and efficacious dose of an anti-EGFR antitumor monoclonal antibody. The data demonstrates that AAVrh10.CetMab-treated mice exhibited a reduction in cachexia, a significant reduction in tumor growth and a prolonged survival following simultaneous and post-xenograft delivery, including a model that makes use of a recently derived GBM patient tumor.

### Gene Therapy for Glioblastoma

The challenge for anti-GBM gene therapy is to develop a safe platform that delivers an efficacious dose of an anti-tumor genetic code for a GBM therapeutic antibody to the GBM microenvironment [35, 36]. Biological vectors, either cells or viruses, are the most common gene therapy delivery tools for GBM [37, 38]. Initial strategies in the early 1990s used replication defective retrovirus or adenovirus to deliver the herpes simplex virus type 1 thymidine kinase gene to the tumor [38–41]. In this cytotoxic suicide gene therapy approach, expression of thymidine kinase phosphorylates the systemically injected prodrug, ganciclovir (GCV), generating the toxic metabolite, GCV-triphosphate [41–43]. Various versions of this strategy have been developed using alternative viral vectors, genetically modified cells and alternative therapeutic genes including cytosine deaminase conversion of the prodrug, 5-fluourcytosine to the toxic metabolite 5-fluorouracil [35, 36, 44]. As an alternative to cytotoxic gene therapy, replication competent oncolytic viruses have been developed to transfer genes that induce tumor cell lysis and activate the host immune response [35, 36, 45]. Oncolytic herpes simplex virus, conditionally-replicating adenovirus, measles virus, vaccinia virus and polio/rhinovirus recombinant virus have been genetically engineered with limited replication in non-cancerous cells and modified tropism for expression in GBM tumor cells [46–49]. While this approach has shown promise in clinical trials, an important caveat to this strategy is that while activation of the host immune system is important for tumor regression, it can also eliminate the cells with therapeutic transgene faster than the oncolytic impact on tumor growth [35, 36].

In contrast to these strategies, in the present study, the therapeutic gene (cetuximab) is targeted to normal brain cells which generate and secrete the anti-EGFR monoclonal which functions in the extracellular milieu to attack the GBM. Using AAVrh10, which has a strong safety profile as a neuronal-specific gene transfer vector, the soluble anti-EGFR antibody distributes into the local microenvironment reducing EGFR-driven GBM growth [24–26] (clinicaltrials.gov, NCT01161576).

### Adeno-associated Delivery of Monoclonals to the CNS

AAV vectors for tropism to the CNS have been widely developed for the treatment of CNS disorders [50–54]. More than a hundred AAV serotypes and variants have been isolated from human and non-human primates, and several have been used in experimental animal studies focused on CNS disorders [55, 56]. Serotypes AAV2, 9 and rh.10 have all been used in human clinical trials directed toward CNS disorders [26, 54, 57] (clinicaltrials.gov, NCT02122952, NCT01161576). Beyond naturally occurring vectors, genetically engineered AAV serotypes also show promise for CNS delivery [58–64]. In the current strategy, we administer AAVrh.10 to the targeted region around the tumor to genetically modify neurons to deliver an efficacious, sustained therapeutic dosage of a soluble monoclonal antibody, circumventing the blood brain barrier that restricts access of systemically administered monoclonal antibodies [24].

The strategy to treat GBM is based on the use of antibodies with high affinity to bind and block activation of cell surface receptors that drive GBM cell growth and proliferation [58, 60]. Monoclonals with high affinity to GBM oncogene drivers have been genetically engineered as non-immunogenic isoforms, such as the humanized bevacizumab and chimeric cetuximab [58, 65–68]. Systemic administration of recombinant monoclonals, including bevacizumab directed against angiogenesis and cetuximab directed against proliferation have demonstrated reduced GBM growth and increased survival in animal models, but the systemic administration of these monoclonals have not increased overall life expectancy in clinical trials [58, 65–68]. Because the human blood brain barrier is considered less permeable than animal models, it is unlikely that an effective dosage of therapy crosses the blood brain barrier into the tumor microenvironment [69]. The systemic mode of delivery and whole-body distribution of the monoclonal drug to non-targeted healthy tissues obviates simply increasing the dose because of the resulting adverse effects [24].

To circumvent this issue, the strategy using cetuximab gene therapy to the CNS could effectively deliver a gene that encodes a long-term effective dosage to the tumor microenvironment, circumventing the blood brain barrier. In the present study, we establish that the AAV vector mediated delivery and expression of cetuximab prolongs survival in human GBM mouse models with U87MG cell line modified to express the EGFR when vector and cells are administered concurrently or when vector is administered after the tumor cells are administered, modelling a therapeutic intervention. Immune compromised mice treated with AAVrh10.CetMab after implanting human primary GBM cells and recently derived patient tumor cells extended survival over control PBS-treated mice. Together, the data support the concept of vector mediated monoclonal therapy directly to the CNS for the treatment of GBM.

## Supporting Information

S1 Fig Change in weight (g) of mice overtime (days post-xenograft) treated with AAVrh.CetMab at the same time as tumor implantation. (PDF)

S2 Fig MRI of mice with U87MG:wtEGFR human glioblastoma xenografts treated 8 days after xenograft implementation. (PDF)
