# Supplementary material for: Anti-Epidermal Growth Factor Receptor Gene Therapy for Glioblastoma
Source: PLoS One. 2016 Oct 6;11(10):e0162978. doi: 10.1371/journal.pone.0162978 (PMC5053413; doi:10.1371/journal.pone.0162978)
Supplement: S1 Fig — (PDF) [file pone.0162978.s001.pdf]

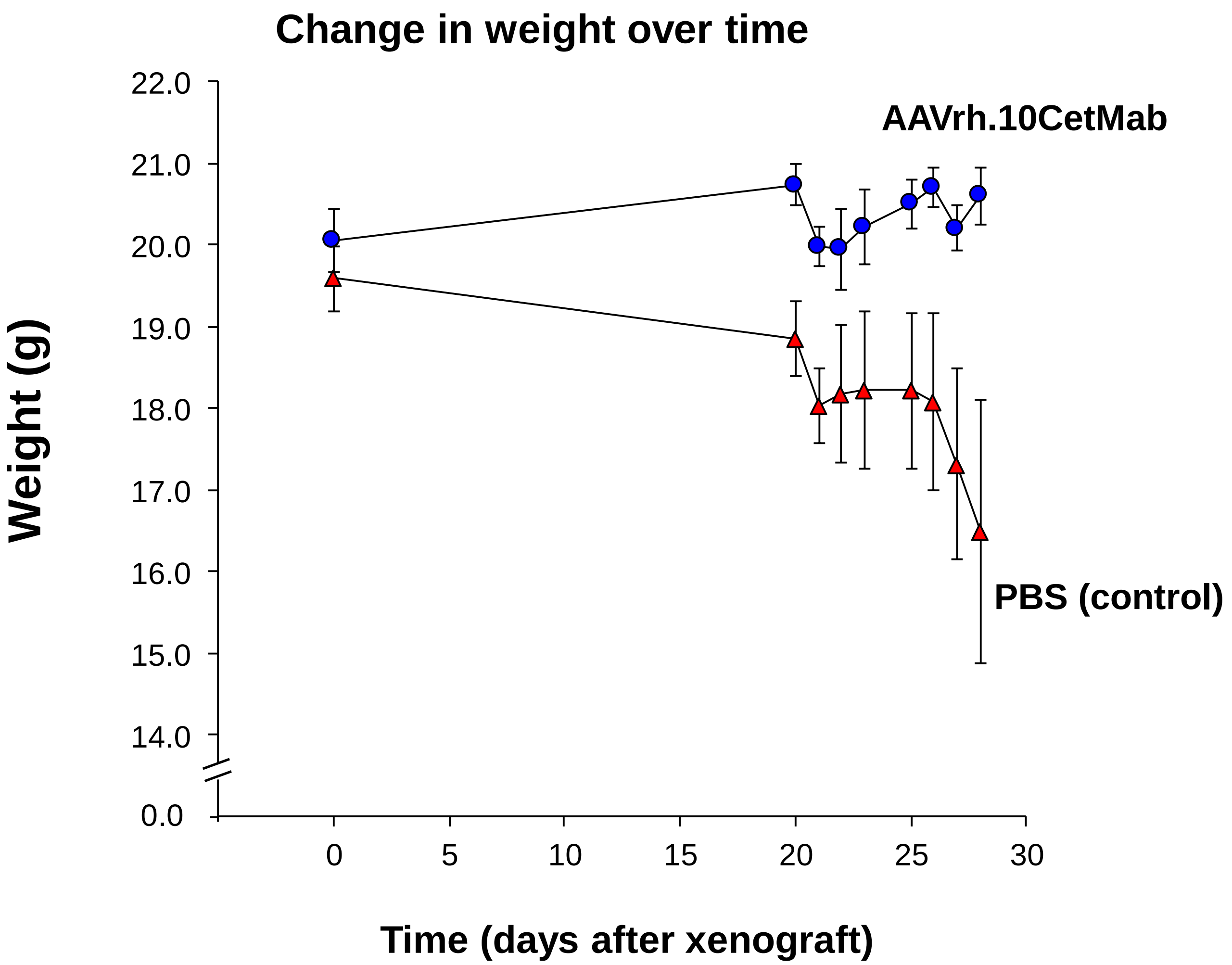

Supplementary Figure 1. Change in weight (g) of mice overtime (days post-xenograft) treated with AAVrh.CetMab at the same time as tumor implantation. NOD/SCID mice (n=6, male) received a single CNS administration of 10^{11} genome copies (gc) of AAVrh.10CetMab or PBS simultaneously with 10^5 U87MG:wtEGFR glioblastoma cells.
