# Supplementary material for: Anti-Epidermal Growth Factor Receptor Gene Therapy for Glioblastoma
Source: PLoS One. 2016 Oct 6;11(10):e0162978. doi: 10.1371/journal.pone.0162978 (PMC5053413; doi:10.1371/journal.pone.0162978)
Supplement: S2 Fig — (PDF) [file pone.0162978.s002.pdf]

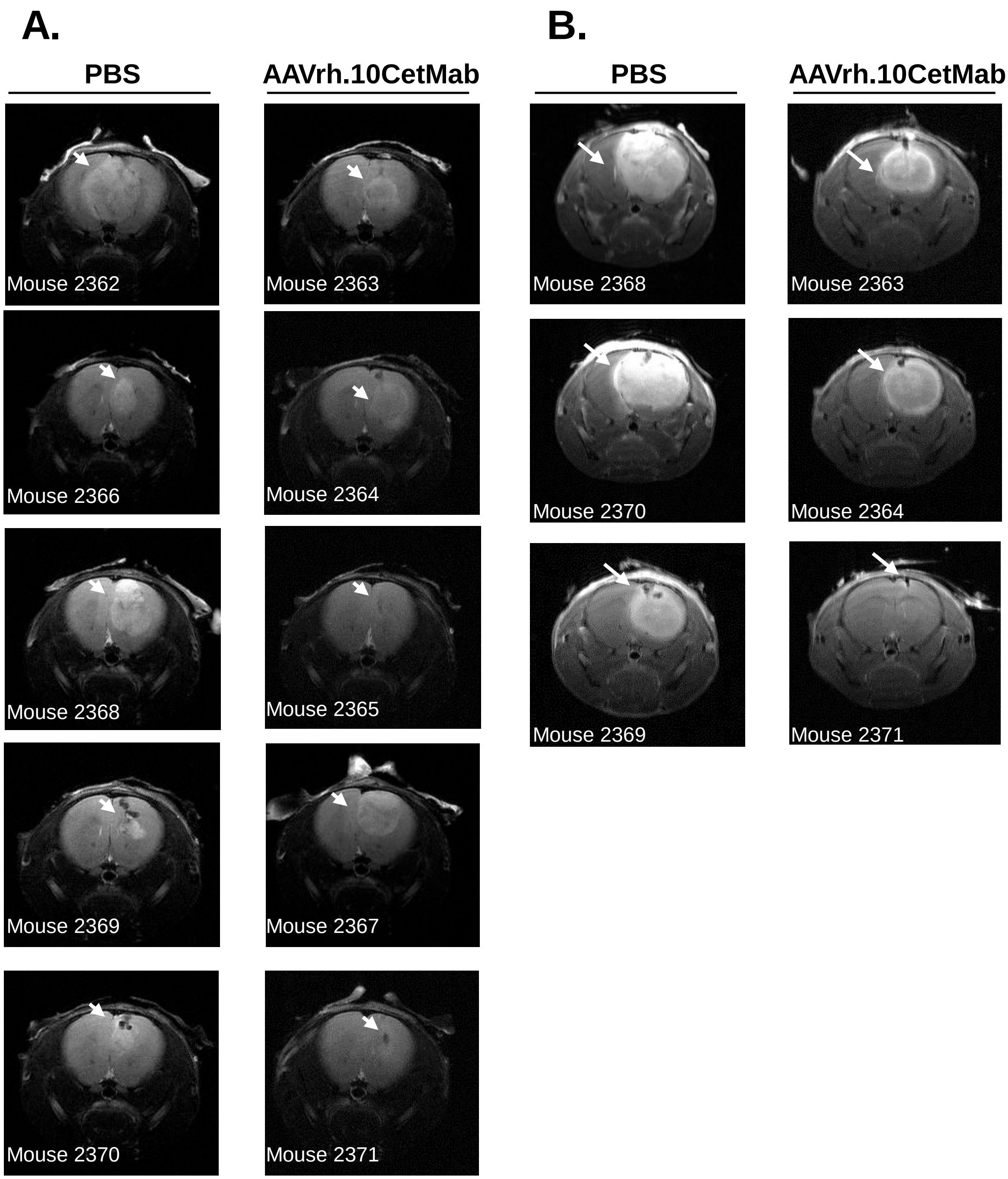

Supplementary Figure 2. MRI of mice with U87MG:wtEGFR human glioblastoma xenografts treated 8 days after xenograft implementation. Male NOD/SCID mice received CNS administration of 10^5 U87MG:wtEGFR glioblastoma cells. Eight days after xenograft implant, mice received 10^{11} (gc) of AAVrh.10Cetmab or PBS. MRI imaging of the tumors was carried out at 3 and 4 wk after treatment administration. **A.** MRI scans at wk 3 after U87MG:wtEGFR implantation, PBS-treated control mice (n=5) and AAVrh.10CetMab-treated mice (n=5). On day 27, two of the PBS-treated control mice (2362 and 2366) were sacrificed due to signs of neurological impairment, cachexia, and significant loss of weight (decrease in 1/3 adult body weight). **B.** MRI scans at wk 4 after U87MG:wtEGFR implantation, PBS-treated control mice (n=3) and AAVrh.10CetMab-treated mice (n=3). Arrows indicate site of tumor on representative coronal image of striatum. Each coronal MRI image corresponds to site of xenograft implantation in distinct mice.
